# Complete Genome Sequences of *Bacillus* Bacteriophages Wes44 and Carmen17

**DOI:** 10.1128/MRA.01103-18

**Published:** 2019-03-21

**Authors:** Haley Alder, Madison Himelright, Emma Eisemann, Louise Temple

**Affiliations:** a James Madison University School of Integrated Sciences, Harrisonburg, Virginia, USA; University of California, Riverside

## Abstract

Wes44 and Carmen17 are siphoviruses that infect Bacillus thuringiensis DSM-350. Wes44 contains 42,248 base pairs and 54 predicted genes; Carmen17 contains 41,820 base pairs and 51 predicted genes. The genomes are 95% similar to each other and distantly related to Bacillus cereus bacteriophage PBC1.

## Abstract (precis)

Wes44 and Carmen17 are siphoviruses that infect Bacillus thuringiensis DSM-350. Wes44 contains 42,248 base pairs and 54 predicted genes; Carmen17 contains 41,820 base pairs and 51 predicted genes.

## ANNOUNCEMENT

Bacillus thuringiensis, a member of the Bacillus cereus group, is a spore-forming bacterium commonly used as a pesticide (1). B. thuringiensis is nonpathogenic to humans and therefore safe as a surrogate to isolate phages in an undergraduate research class. Some B. cereus strains cause food poisoning in humans (2) and would be good targets for phages that could be useful in food safety.

**FIG 1:**
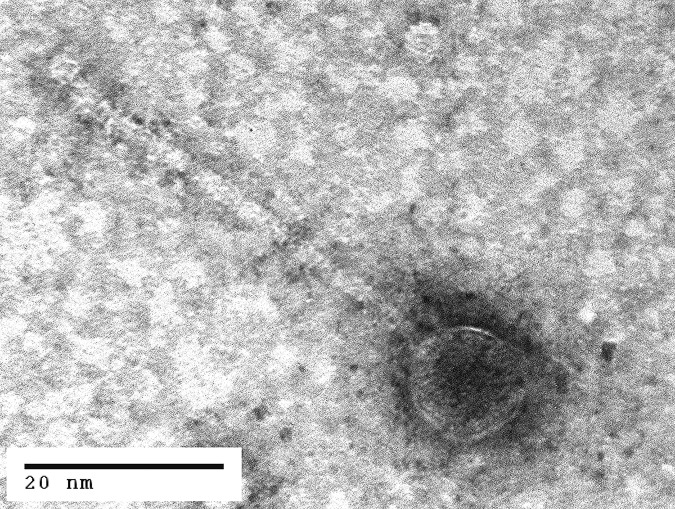
Transmission electron micrograph of a representative Carmen17 phage sample. Wes44 was indistinguishable in similar micrographs. For electron microscopy, phages from lysate were negatively stained with 1% uranyl acetate on Formvar-coated copper grids and photographed on an FEI Morgagni 268 transmission electron microscope (FEI, Hillsboro, OR).

These siphoviruses (Fig. 1) were isolated in September 2015 from soil in Virginia (both 37°05′N, 76°51′W) by first enriching the soil sample with B. thuringiensis and then sterilizing and plating on the host. Phages were purified using standard microbiological techniques, and genomic DNA was isolated using a modified ProMega kit protocol (3). The DNA was sequenced to >60-fold coverage by the Genomic Sequencing Lab at North Carolina State University. For each genome, 50,000 randomly derived reads of raw data (Command: head −n 50000 completedataset.fastq > newfilename.fastq) were assembled into a single contig using Newbler 2.1 (4). Genes were predicted using GeneMark.hmm version 1 (5) and Glimmer 2.1 (6) and annotated using DNA Master (7).

Using BLASTn analysis (8), we found that Wes44 and Carmen17 were 95% similar to each other and shared 49 core genes. All predicted genes were transcribed in the same direction. The closest relative among phages in GenBank was B. cereus phage PBC1 (9, 10), isolated in South Korea in 2012, which had 76% DNA identity over 49% of the genome. Phage PBC1 contained 40 of the 49 core genes from Wes44 and Carmen17, and the genomes were colinear. Morphologically, Wes44 and Carmen17 had flexible tails ∼50 nm in length and heads ∼15 nm in diameter (Fig. 1).

Wes44 and Carmen17 had circularly permuted DNA, suggesting headful packaging. Wes44, Carmen17, and PBC1 each had a G+C content of ∼42%, contrasting with that of their hosts, which had G+C contents of ∼35%. This difference might indicate that these are not the optimal hosts for these phages. In contrast to PBC1, Wes44 and Carmen17 consistently formed very cloudy plaques; however, no genes indicating a lysogenic lifestyle were found. Therefore, the phages have not been designated either virulent or temperate.

Sequence similarity searching with BLASTp (8) revealed packaging and structural proteins (terminase large and small subunits, a portal protein, major and minor capsid proteins, and a tail length measure protein), a holin and an endolysin, and DNA replication and modification proteins (thymidylate synthase, nucleoside triphosphatase, DNA polymerase, a glutaredoxin-like protein, a nuclease, a helicase, and others). Like PBC1, these phages had a predicted YD repeat protein implicated in carbohydrate binding (10), which could explain the very limited host range, as reported for PBC1 (10). Two B. cereus strains (ATCC 14579 and FDA4) were tested for sensitivity to Wes44 and Carmen17, and no infection was evident using approximately 2 × 10^5^ PFU. The predicted endolysin from the two phages was highly similar (60% amino acid identity) to the related endolysins from PBC1 and phage 12826, which have been tested as a detection method for B. cereus (10).

PBC1, Wes44, and Carmen17 will constitute a new *Bacillus* phage cluster, which has not yet been named.

### Data availability.

This whole-genome shotgun project has been deposited in DDBJ/ENA/GenBank under the accession numbers MG784342 for Carmen17 and MH598512 for Wes44. The short read sequences have been deposited under BioProject number PRJNA515721 and Sequence Read Archive number SRP182825.
